# Efficacy of removing *Candida albicans* from orthodontic acrylic bases: an in vitro study

**DOI:** 10.1186/s12903-019-0765-x

**Published:** 2019-05-02

**Authors:** Abdul Razzak A. Ghazal, Ghassan Idris, Mohammad Y. Hajeer, Karam Alawer, Richard D. Cannon

**Affiliations:** 1 Technical Institution of Dentistry, University of Hama, Hama, Syria; 2 Sir John Walsh Research Institute, University of Otago, Dunedin, New Zealand; 3 Department of Orthodontics, University of Damascus Dental School, Damascus, Syria; 4 Research microbiology Laboratory, Hama University, Hama, Syria

**Keywords:** *Candida albicans*, Orthodontic acrylic, Orthodontic appliances, Disinfection

## Abstract

**Background:**

This study evaluated the efficacy of four methods in removing *Candida albicans* from the acrylic base material used to fabricate removable orthodontic appliances.

**Methods:**

Heat-processed bars of orthodontic acrylic were incubated in a suspension of *C. albicans* for 2 h at 37 °C. Samples were allocated into five groups (five bars per group) according to the cleaning method: (1) manual brushing using a toothbrush; (2) soaking in a commercial denture cleaning solution; (3) soaking in a commercial mouthwash solution; (4) using an ultrasonic cleaner; and (5) soaking in distilled water as a negative control. Yeast remaining attached to the bars after cleaning were removed by vortexing in growth medium and plated on Sabouraud dextrose agar. The reduction in yeast colony count after cleaning was calculated and expressed as the number of colony forming units per acrylic bar (CFU/bar). The experiment was carried out three times.

**Results:**

All four cleaning methods resulted in a significant decrease in viable yeast cells associated with the acrylic bars compared to the control group. The mean percentage reduction in viable yeast cells affected by the cleaning methods was: brushing 89.9%; chlorhexidine 95.8%; ultrasonic cleaning 99.9%; and denture tablet 100%.

**Conclusions:**

All four methods evaluated in this study were effective, to some extent, in removing *C. albicans* from orthodontic acrylic samples. The most effective, and readily available, cleaning method was the use of commercial denture sterilizing tablets.

## Background

*Candida albicans* is considered one of the normal components of the oral flora as more than 60% of healthy populations are colonized by this yeast without clinical symptoms of infection [1]. *C. albicans,* which is the most frequently isolated species of *Candida* [2], could pose a serious clinical challenge in immune-compromised individuals (people with HIV infection, xerostomia, diabetes, leukemia etc.) [3–5]. There is an increasing number of children surviving cancer who, just like their healthy peers, seek orthodontic treatment [6]. This is a special group of patients that should be particularly motivated to maintain dental hygiene [6, 7], because the reduction in immune function allows *C. albicans* to proliferate and to cause infection [8, 9]. Similarly, children whose saliva contains reduced amounts of antimicrobial proteins have less protection from candidiasis [10].

Removable orthodontic appliances are a popular tool to move, or retain, teeth during, or after, orthodontic treatment [11, 12]. Nocturnal wearing of the acrylic removable orthodontic appliances may play a role in the development of oral *C. albicans* infections. This could be due to low salivary flow and consequently low pH levels as well as impaired oral hygiene [8, 13, 14]. Wearing acrylic orthodontic appliances has been reported to be associated with increased proliferation of *Candida* regardless of the host immune system status [2, 11–13]. These appliances provide an enhanced environment for *C. albicans* growth as they cover a large area of mucosal tissues for a considerable amount of time every day for a relatively long period [15, 16]. The removable appliances protect the yeast from the natural flow of saliva and mechanical removal effects of musculature. The orthodontic acrylic also provides a hydrophobic surface to which *C. albicans* can bind through the hydrophobic effect and van der Waals forces. [14]. The combination of these factors may tip the balance in the wearers of orthodontic appliances to support *Candida* colonization and proliferation [2, 8] and also increase dentine demineralization by enhancing the cariogenic potential of *Streptococcus mutans* containing biofilms [17].

Although the increased colonization of individuals wearing removable dentures and removable orthodontic appliances by *C. albicans* might be expected to be similar [18], the two types of acrylic resin have different physical properties, and different fabrication and polymerization methods that affect the amount of residual monomer, which might affect the surface morphology and *C. albicans* adhesion [12, 19, 20].

Several in vitro studies have shown that different cleaning methods remove *C. albicans* from acrylic removable dentures to different extents. These studies have compared the efficiency of using chemical and mechanical methods of removing *C. albicans* from acrylic denture base resin [21–23], but there is no published data evaluating the procedures used in cleaning removable acrylic orthodontic appliances. The aim of this research was to assess the effectiveness of brushing with a toothbrush, soaking in a solution of a commercial denture cleaner, immersing in chlorhexidine gluconate oral rinse solution and using an ultrasonic cleaning to remove *C. albicans* from orthodontic acrylic resin.

## Methods

### Fabrication of orthodontic acrylic samples

Seventy-five identical acrylic bars with a surface topology of clinical relevance were fabricated using duplicated dental stone casts of an 11 year-old orthodontic patient. An alginate impression (Hydrogum®, Zhermack, Badia Polesine, Italy) of the upper dental arch of the patient was taken as part of routine orthodontic treatment, and a cast made using yellow dental stone (Maarc™, Mumbai, India).

**Fig. 1:**
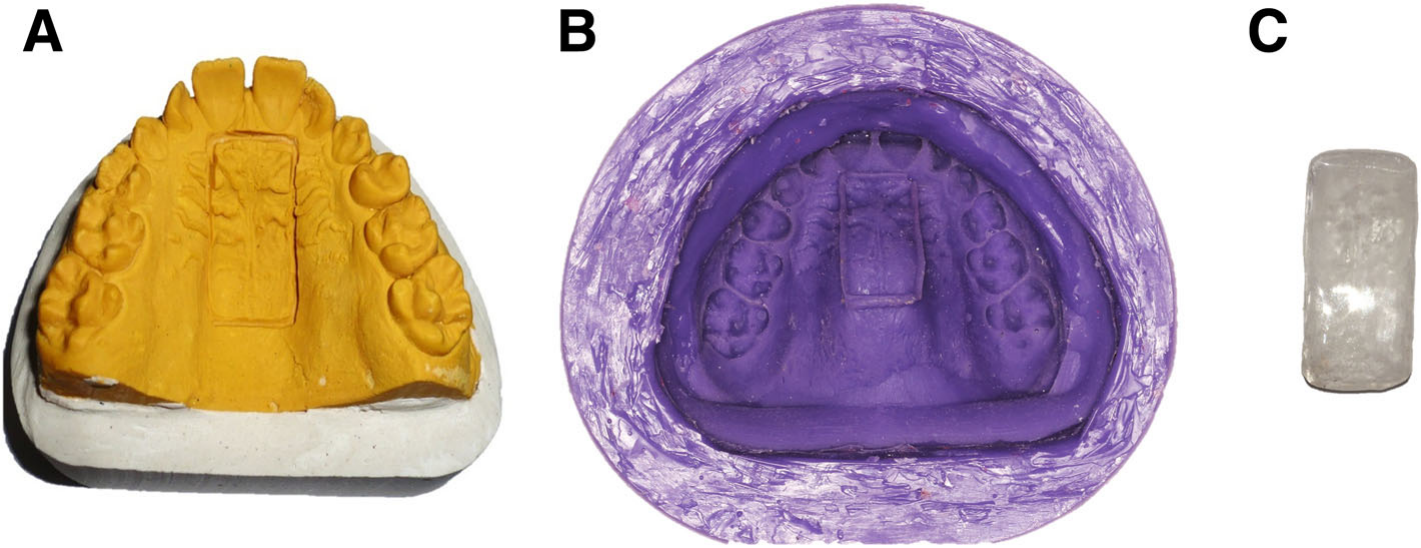
Fabrication of orthodontic acrylic bars: **a** Dental cast with 12 × 25 × 2 mm rectangular sulcus carved in the palate, **b** The silicon mold used to fabricate stone models and **c** One of the acrylic bars

In order to fabricate identical acrylic bars, the following procedure was applied to the cast. A rectangular sulcus (12 × 25 × 2 mm) was carved in the palate portion of the dental cast (Fig. 1) and the modified cast was copied using silicon duplication material: Silicopy, SPD, Mulazzano, Italy.

Using this silicon mold, 75 yellow stone casts were poured (in three groups of 25 casts). To fabricate the acrylic bars, the spray-on (salt and pepper) method was used to fill in the marked rectangular area in each cast using an orthodontic heat-polymerized acrylic resin (Dentaurum Orthocryl®, Ispringen, Deutschland). The dimensions of the bars were confirmed using a Boley Gauge Caliper. The heat-polymerization process was used for all the samples according to manufacturer’s instructions. The acrylic bars were finished and polished on only one side to simulate the fabrication of removable orthodontic appliances (Fig. 1).

### Isolation and identification of *Candida albicans*

Fresh *C. albicans* clinical isolates were obtained by sampling the palatal surface of a patient’s removable orthodontic appliance with a sterile swab, plating on Sabouraud dextrose agar with chloramphenicol (HiMedia®, Mumbai, India) and incubating at 37 °C for 48 h.

Isolates were confirmed as *C. albicans* by the germ tube test and chlamydospore formation as described previously [14, 24].

### Coating acrylic bars with human saliva

Acrylic bars were coated with human saliva to mimic the in vivo condition for orthodontic appliances. Unstimulated saliva (10 mL) was collected from each of 12 healthy adults (6 males and 6 females), mixed and centrifuged at 5000×g for 10 min at 4 °C [25]. The saliva supernatant was immediately stored at − 70 °C until use.

### Ethical approval and consent forms

Following approval of the study by the University of Hama Local Ethics Committee, and providing verbal and written explanation of the intended research, consent forms were signed by the parents of the patient whose orthodontic impression was used and by those who provided unstimulated saliva samples.

### Preparation of *C. albicans* to coat acrylic

The *C. albicans* clinical isolate was cultured on Sabouraud dextrose agar with chloramphenicol (HiMedia®) at 37 °C for 48 h. A yeast suspension was prepared by harvesting *C. albicans* cells and adjusting the concentration with dual distilled water to 10^7^ CFU/mL (colony forming unit/mL) by comparing it with the optical density of a McFarland standard [22, 23].

### Attachment of *C. albicans* to acrylic bars

The acrylic bars were disinfected by immersing them in alcohol 70% *v*/v (volume per volume) for 10 min and then washing them in ultrapure water for 1 min. Next, bars were individually exposed to 2 ml of the previously prepared human saliva in test tubes at room temperature for 30 min [26, 27] and washed in ultrapure water for 1 min. Each acrylic bar was then placed in a test tube containing 8 mL of the *C. albicans* suspension and placed in an incubator at 37 °C.

**Fig. 2:**
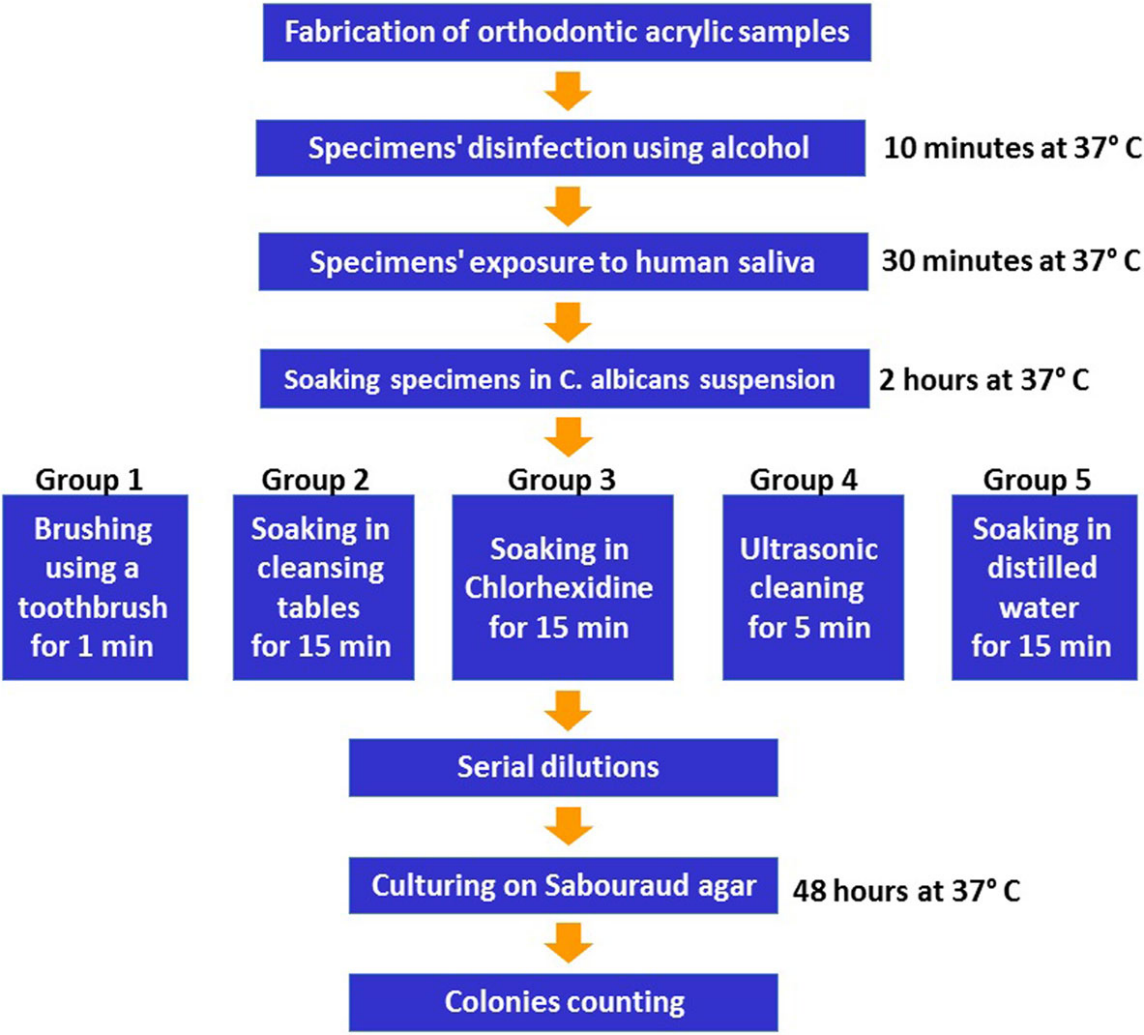
Flow chart of the method employed in the study

After 2 h incubation, each sample was removed using sterile tongs, washed with 5 mL of ultrapure water (Fig. 2).

### Cleaning of specimens

All disinfection assays were performed on groups of five acrylic bars (*n* = 5). The assays were carried out on three separate occasions with freshly grown *C. albicans* cells. The following procedures were applied to the groups of five bars (Fig. 2).

The Brushing group (Group 1) samples were brushed manually using a medium-bristle toothbrush (123 Classic Care, Oral-B®, UK) on all sides under running sterilized water for 1 min. A new toothbrush was used for each bar.

The Tablets group (Group 2) samples were immersed in 200 mL of ultrapure water in which one tablet of commercial denture cleaner (Corega® tabs – GlaxoSmithKline, Ireland, UK) had been dissolved, for 15 min at room temperature. According to the manufacturer, the tablets contained: sodium bicarbonate, citric acid, potassium monopersulfate, sodium carbonate, sodium carbonate peroxide, tetra-acetyl ethylene diamine, sodium benzoate, PEG-180, sodium lauryl sulfoacetate, PVP/VA copolymer, aroma, subtilisin, sodium nitrite, CI 42090 (Brilliant Blue colorant) and CI 73015 (Indigo carmine colorant).

The Chlorhexidine group (Group 3) samples were immersed in 200 mL of 0.12% of chlorhexidine gluconate (Peridex™: 3 M, MN, USA) for 15 min at room temperature .

The Ultrasonic cleansing group (Group 4), samples were cleaned using an ultrasonic cleaner (CD-4820, GS, China) at a frequency of 50 Hz and power of 170 W for 5 min.

The last group was the control group (Group 5) and samples were immersed in 200 mL of ultrapure water for 15 min.

### Assessment of efficacy of the cleaning methods

Subsequent to the cleaning treatment, samples were placed in 1 mL of Sabouraud dextrose with chloramphenicol broth and vortexed for 2 min. Tenfold serial dilutions of each sample were prepared from 10^1^ to 10^5^, then 100 μL from each dilution was spread on a Sabouraud dextrose agar plate. The agar plates were incubated at 37 °C for 48 h. The *C. albicans* colonies on the agar plates were counted manually (the total number of viable *C. albicans* cells = the number of colonies x the dilution factor × 10) and expressed in number of colony forming units per millilitre (CFU/mL) which is equivalent to the CFU/bar (Table 1). The percentage reduction due to the cleaning was calculated using the following formula: persentage reduction = (CFU/bar_c_ – CFU/bar_t_) ÷ CFU/bar_c_ × 100 where CFU/bar_c_ = CFU/bar with no treatment (control) and CFU/bar_t_ = CFU/bar after treatment.

**Table 1:** Effect of different cleaning methods in the removal of *C. albicans* from acrylic bars

Treatment method	CFU/bar, mean ± SD (range) *n* = 3		Reduction %	Statistical analysis, *P* values	
	CFU/bar with no treatment (Control)	CFU/bar after treatment		Comparison with control	Inter-treatment comparison
Brushing	9133 ± 3282 (4300–15,100)	918.7 ± 267.7 (620–1420)	89.9	< 0.01	To Tablet > 0.05 (0.092) To CHX > 0.05 (0.323) To US > 0.05 (0.095)
Tablets		0 ± 0 (0–0)	100	< 0.01	To CHX > 0.05 (0.479) To US > 0.05 (0.990)
CHX		382.7 ± 131.1 (180–620)	95.8	< 0.01	To US > 0.05 (0.487)
US		6.67 ± 11.13 (0–30)	99.9	< 0.01	N/A

*CHX* chlorhexidine, *US* ultrasonic cleaner

### Statistical analysis

Since the data of all groups were normally distributed (Anderson-Darling test), parametric tests were used. Because the experiment was conducted three times, repeated measures analysis of variance (rANOVA) was used to assess any discrepancy in the results obtained in the three replicates of each method. With alpha set at 5%, one-way analysis of variance (ANOVA) was used to detect differences in the CFU/ml counts between the five groups. This was followed by least significant difference (LSD) post hoc pairwise comparisons. All analyses were conducted using SPSS software (SPSS, IBM Corp. Version 22.0. Armonk, NY, USA).

## Results

The *C. albicans* clinical isolate adhered well to the saliva-coated orthodontic acrylic bars, with an average of 9133 cells recovered from the bars by vortexing in growth medium. All cleaning methods reduced the number of *C. albicans* CFU adhered to the bars (Table 1). Repeated measures ANOVA showed no significant differences between the three replicates in Brushing group (*P* = .850), Tablets group (equal values), Chlorhexidine (*P* = .416), Ultrasonic group (*P* = .605) or Control group (*P* = .371).

The different cleaning methods reduced the number of *C. albicans* CFU adhering to orthodontic acrylic bars to differing extents. The order of efficacy of removing *C. albicans* CFU was: Tablets > Ultrasonic cleansing > Chlorhexidine > Brushing. There were no detectable viable *C. albicans* cells associated with the acrylic bars after treatment with denture Tablets. Regarding the differences observed between the five groups, LSD *post-hoc* tests showed that there was a significant difference between the control group compared to the other groups (Table 1) (*P* < 0.001). Pairwise comparisons of brushing, tablets, chlorhexidine and ultrasonic groups showed no significant difference between cleaning methods (Table 1).

## Discussion

Acrylic resin plates are widely used as removable orthodontic appliances and it is important to understand the effect of wearing orthodontic appliances on intraoral colonization by bacteria and fungi. Removable orthodontic appliances are important tools in the orthodontist’s armamentarium and they are commonly use in treating a range of malocclusions. The use of such appliances has been shown to inhibit oral hygiene occlude surfaces and enhance the proliferation of microorganisms [11, 28].

*C. albicans* is considered a significant opportunistic pathogen due to its ability to adhere to a variety of surfaces, generate drug-resistant biofilms, and secrete proteinases and a toxin [29]. These numerous virulence factors mean that if it’s growth is not kept in check it can become pathogenic both locally and systemically leading to various forms of candidiasis [29, 30]. The importance of keeping removable orthodontic appliances appliances *Candida*-free has been emphasised in several reports [11, 13, 15], however, this study is the first to compare the effectiveness of different methods of removing *C. albicans* cells from these widely used appliances.

This study used clinically relevant replicates of orthodontic acrylic bars reproducibly coated with a relatively large number of *C. albicans* cells. The cells probably adhered to the micro-indentations on the internal non-polished surface of the acrylic as with dental acrylic [31]. The porosity and water-absorbing ability of heat-cured acrylic makes an amenable environment for *C. albicans* colonization and growth [32].

Brushing with a toothbrush, which is considered the most common method of controlling plaque development on acrylic surfaces [33], removed 89.9% of *C. albicans* cells. A similar finding was reported by Pellizzaro et al. [34] who obtained a 96% reduction of *Candida* biofilm. Other studies have shown that brushing was an ineffective method for *Candida* removal [35, 36]. Contrary to the findings presented here, a study comparing mechanical brushing to chemical methods found that a toothbrush is more effective than some chemical methods when using cleaning tablet. [37]. This difference could be explained by the use of dentifrice with the toothbrush in the study by Paranhos et al. [37] and soaking samples in tablets for a shorter time (i.e., 5 min). It is possible that the toothbrush is unable to dislodge cells within the pores of the fitting surface of the acrylic bars.

Soaking acrylic samples in chlorhexidine 0.12% caused a significant 95.8% reduction in viable cells associated with the acrylic bars. This is due to the fact that chlorhexidine is able to increase *C. albicans* cell permeability allowing the chlorhexidine gluconate to enter the cell [38], leading to cell wall damage and cell death [39]. Immersing acrylic appliances in chlorhexidine for a relatively short time mimics the real life use of mouthwashes which have been shown to be effective against *Candida* [38, 40–42]. Mouthrinses have an extra advantage of their pleasant odor [40] that might encourage routine disinfection of removable orthodontic appliances.

Ultrasonic cleaning of the acrylic bars was an effective method that removed more than 99.9% of *C. albicans* cells. Kawasaki et al. [31], however, reported that more than 11% of *Candida* cells remained following 15 min of ultrasonic cleaning (even though the maximum removal was achieved in the first 5 min). The reduced effectiveness of ultrasonic cleaning in that study could be due to the different acrylic base resin, *C. albicans* strain and ultrasonic cleaner used. Muscat et al. suggested the use of an ultrasonic treatment for only 30 s as being an effective method for removing *C. albicans*, but the acrylic samples were made of self-cure poly methyl methacrylate (PMMA). Although effective in the present study, it is unlikely that ultrasonic cleaners will be widely used by orthodontic patients for cleaning their removable appliances.

Soaking the orthodontic acrylic bars in a commercial denture cleaning solution (dissolved tablets) resulted in no viable *C. albicans* cells associated with the bars. Many researchers [23, 34, 40] have used effervescent tablets to clean acrylic dentures since they are promoted as acrylic-friendly products even when used for prolonged periods [43]. The mechanism of cleansing acrylic resin with tablets starts once the tablet dissolves in water, as an alkaline peroxide solution is formed. This solution then releases oxygen bubbles enabling a mechanical cleaning [23] in addition to chemical cleaning enhanced by sodium lauryl sulfoacetate, a detergent that penetrates through the cell wall and permeabilises the cell membrane causing leakage of intracellular components and cell lysis [34, 44]. These multiple mechanisms may be responsible for eliminating all viable *C. albicans* from orthodontic acrylic specimens in the current study.

Montagner et al. reported that cleansing tablets were ineffective when soaking acrylic samples for only 5 min [45] while Yildirim-Bicer et al. [23] found that 0.1% of *Candida* cells survived after 10 min soaking. However, in the current study the acrylic bars were soaked for 15 min, this additional soaking time could have increased the efficacy of sample disinfection. Iseri et al. [40] found that tablets could not completely eliminate *Candida* from denture base acrylic resin even after 60 min disinfection. However, this study used tablets with a different chemical composition that did not contain sodium lauryl sulfoacetate which may be the efficacious component for *C. albicans* elimination. [34]. It should be noted that in the current in vitro study, orthodontic acrylic bars were coated with only *C. albicans* - without any other microorganisms. In the oral cavity, *C. albicans* will be incorporated into poly-microbial communities on orthodontic acrylic appliances leading to a complex interaction that could modulate *Candida* adherence and reduce the efficacy of the cleansing methods evaluated in the current study [46].

## Conclusions

This study showed that brushing, the denture cleaning tablets, chlorhexidine gluconate and ultrasonic cleaning can remove *C. albicans* from the surface of orthodontic acrylic. Although no significant difference was found between the test methods, commercially available denture cleaning tablets proved to be the most effective method.

## Abbreviations

*C. albicans*: *Candida albicans*
CFU: Colony forming unit
CHX: Chlorhexidine
LSD: Least significant difference
PMMA: Poly methyl methacrylate
rANOVA: Repeated measures analysis of variance
US: Ultrasonic
v/v: Volume per volume
μL: Microliter
